# Mini-Invasive Bentall Procedure Performed *via* a Right Anterior Thoracotomy Approach With a Costochondral Cartilage Sparing

**DOI:** 10.3389/fcvm.2022.841472

**Published:** 2022-03-02

**Authors:** Qiang Ji, YuLin Wang, FangYu Liu, Ye Yang, Jun Li, XiaoNing Sun, ZhaoHua Yang, Sun Pan, Hao Lai, ChunSheng Wang

**Affiliations:** ^1^Department of Cardiovascular Surgery, Zhongshan Hospital, Fudan University, Shanghai, China; ^2^Shanghai Municipal Institute for Cardiovascular Diseases, Shanghai, China

**Keywords:** aortic root replacement, Bentall’s procedure, minimally invasive cardiac surgery, right anterior minithoracotomy approach, costochondral cartilage sparing

## Abstract

**Objectives:**

A right minithoracotomy approach with a sternal sparing technique is a minimally invasive option for surgeons performing aortic root surgery. This report presents our initial clinical results of the right minithoracotomy Bentall procedure.

**Methods:**

Clinical data of 15 patients were retrospectively analyzed who underwent the minimally invasive Bentall procedure through the right anterior thoracotomy *via* the second intercostal incision without any costochondral cartilage invasion at our institution between October, 2019 and June, 2021. The operative time, length of intensive care unit stay and postoperative hospital stay, perioperative outcomes, and follow-up results were analyzed.

**Results:**

The median aortic cross-clamping time was 95.0 (85.5–98.8) min. Three (21.4%) patients received blood transfusion. The median drainage volume in the first 24 h was 200.0 ml, with no redo for bleeding. The median duration of mechanical ventilation was 12.5 (11.0–25.0) h, and median length of intensive care unit stay was 1.5 (1.0–3.0) day. All patients discharged 5.8 ± 1.2 days following surgery, with no dead patients found. At 6 months following surgery, all patients survived with an improved New York Heart Association (NYHA) functional class.

**Conclusion:**

The right minithoracotomy Bentall procedure may be performed safely with low morbidity and mortality. This approach should be considered as an option in carefully selected patients requiring aortic root replacement.

## Introduction

As technology advances and surgeon experience increases, increasing patients undergo mini-invasive cardiac surgery, and the range of minimally invasive cardiac surgery continues to broaden. The most common surgical approach to aortic root replacement is a full median sternotomy (1, 2). An increasing number of surgeons are employing a ministernotomy approach, including upper hemi-sternotomy and other partial minimal sternal variations, to complete this procedure (3–5). The ministernotomy approach confers advantages with respect to bleeding, respiratory recovery time, and risk of mediastinitis when compared with full sternotomy approach in selected patients (3–5).

A right anterior minithoracotomy approach for minimally invasive aortic valve replacement (AVR) is a well-established surgical procedure. It has several advantages over AVR through sternotomy in terms of decreased blood loss, shortened length of hospital stay, decreased pain, early recovery of pulmonary function, improved cosmesis, and a rapid return to daily activities (6, 7). The success of the right minithoracotomy AVR may translate into favorable outcomes in selected patients undergoing the right minithoracotomy approach for aortic root surgery. Recently, a right minithoracotomy Bentall procedure which includes the utilization of video guidance and automated suturing technology has been described (8).

At our institution, the right anterior minithoracotomy approach has been the preferred minimally invasive technique used for isolated AVR. We have recently introduced the right minithoracotomy Bentall procedure with conventional instruments and suturing techniques. In this report, we presented our initial clinical results of right minithoracotomy Bentall procedure, and tried to evaluate its feasibility and safety.

## Materials and Methods

### Patients

Between October, 2019 and June, 2021, clinical data of consecutive patients aged over 18 years who underwent mini-invasive Bentall’s procedure *via* a 6-cm right anterior thoracotomy incision with a costal cartilage sparing technique in this center were reviewed. All included patients suffered from aortic sinus pathology with concomitant aortic valve disease without arch lesions documented by echocardiography. Computed tomography was performed as needed to assess the anatomy of the aortic root and the ascending aorta. Pulmonary function testing was frequently conducted.

### Study Protocol

This study protocol was approved by the Ethics Committee of *Zhongshan Hospital Fudan University* and was consistent with the *Declaration of Helsinki*. All included patients signed an informed consent approved by the ethics committee.

Baseline and surgical characteristics, and perioperative outcomes were obtained retrospectively from our institutional database and were reviewed using a standard data collection form. The operative time was evaluated on the basis of aortic cross-clamping time and duration of cardiopulmonary bypass. The operative characteristics and perioperative outcomes (including surgical death, blood transfusion requirement, redo for bleeding, low cardiac output syndrome, prolonged mechanical ventilation of more than 72 h, new-onset cerebrovascular adverse events, acute kidney injury requiring hemodialysis, and length of ICU stay and postoperative hospital stay) were analyzed. The definitions and variables selected were based on *The Society of Thoracic Surgeons Database* definitions.

Patients were regularly followed up at 1-, 3- and 6-month following surgery and in 6-month intervals thereafter. Follow-up data were obtained through clinic visits or telephone interviews. Follow-up results included survival, reoperation, New York Heart Association (NYHA) functional class, and echocardiographic data.

### Surgical Procedure

Surgery was performed under general anesthesia with double-lumen endotracheal intubation. Transesophageal echocardiography (TEE) and cerebral oximetry were initiated. Soft pads were put under the right side of the patient’s body with an inclination of 15–30°. Defibrillation electrode pads were placed behind the right scapula and on the 5th intercostal space of left anterior axillary line. The patient was prepped from above the clavicles to the knees bilaterally.

A femoral or axillary platform was utilized to establish the cardiopulmonary bypass (CPB). A 2–3 cm incision was made in the inguinal crease preferably on the right side, and a 5–0 Prolene purse-string suture was placed on the femoral or axillary artery and femoral vein. After heparinization, a *Seldinger* technique was utilized to cannulate the femoral/axillary vessels. The femoral/axillary artery was cannulated with a 16–19 Fr arterial cannula, and the femoral vein with a 24 Fr venous cannula. TEE was used to aid placement of the venous cannula in the superior vena cava.

A 6-cm incision (main incision) in the second intercostal space, starting two fingers lateral to the sternal border and extending laterally, was made, and the right pleural space was entered after one-lung (left lung) ventilation. The right mammary artery and vein were not transected, nor was rib or costal cartilage resection performed. Afterward, a soft tissue retractor (LAP Protector FF1210, Hakko Co., Ltd., Nagano, Japan) was inserted, and an intercostal rib spreader (Valve XS, Aesculap, Inc., PA, United States) was used to provide further visualization. Another incision for a 5-mm port (utility port) in the fourth or fifth intercostal space of right middle axillary line was made. After identifying the phrenic nerve, the pericardium was opened over the aorta and extended down toward the inferior vena cava. The pericardial stay sutures were placed and were clamped to the soft tissue retractor.

The CPB was initiated at 32–36°C using a closed-membrane oxygenator and roller pump. Venous drainage was augmented with vacuum assistance, applying a negative pressure of 30–70 mmHg as needed to decompress the right heart. A left ventricular vent passed through the utility port and was placed through the right superior pulmonary vein. *Trans*-incisional direct aortic cross-clamping was performed, utilizing a flexible and retractable shaft cross-clamp (Novare Surgical Systems, Cupertino, CA, United States). Myocardial protection was achieved using a modified *Del Nido* cardioplegia solution (4 parts blood to 1 part cardioplegia) at 4°C *via* direct coronary anterograde perfusion. The initial dose of anterograde cardioplegia infused was at 20 mL/kg. Additional doses will be administered at 90 min.

**FIGURE 1:**
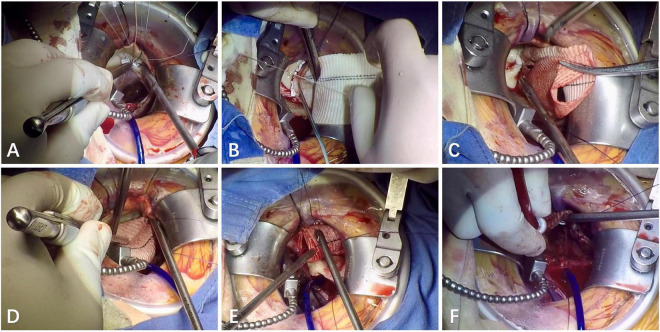
Right minithoracotomy Bentall procedure. **(A,B)** The images show the annular sutures using a double-armed 2–0 poly (ethylene terephthalate) suture with gasket and an interrupted mattress suturing technique; **(C)** the image shows the anastomosis of the left main coronary-graft with a running fashion using 5–0 polypropylene; **(D)** the image shows the anastomosis of the right main coronary-graft with a running fashion using 5–0 polypropylene; **(E)** the image shows the distal anastomosis of graft-native ascending aorta with a running fashion using 4–0 polypropylene; **(F)** the image shows that the left ventricle and the graft were de-aired with a venting needle in the root of the aorta before the cross clamp was removed.

The aortotomy (H-shaped incision) was made at the point of the antegrade needle insertion. The proximal aorta was transected at the level of the sinotubular junction, and the amount of the distal ascending aorta removed was tailored on the basis of the pathology. Three commissural Prolene stay sutures were placed, which allowed exposure and rotation of the aorta toward the field of vision. A gauze pad was placed in the left ventricle temporarily to collect any debris. The leaflets were excised and calcification in the aortic annulus was debrided as necessary. The decision to implant mechanical prosthetic valve or bio-prosthetic heart valve was influenced by each patient’s demographic and clinical profile (i.e., age, peptic ulcer bleeding, the size of the aortic valve annulus). The size of the prosthetic valve was based on intraoperative actual measured values using the valve sizer. It was very important to implant a sufficiently large prosthetic valve in adult patients. In the case of implanting a mechanical prosthetic valve, a vascular graft with a mechanical prosthetic valve (Aortic Valved Graft Medtronic Inc., Minneapolis, United States; SJM Masters Series, St. Jude Medical Inc., St. Paul, MN, United States) was typically used; whereas a vascular graft (Terumo Cardiovascular Group, Tokyo, Japan; MAQUET Cardiovascular, Wayne, NJ, United States) was frequently used and a bioprosthetic valve was sewn into the graft using a running 4–0 polypropylene when bio-prosthetic heart valve was required. The annular sutures were placed with a double-armed 2–0 poly (ethylene terephthalate) suture with gasket and an interrupted mattress suturing technique, beginning with the commissural sutures followed by the left, non-coronary, and right sutures, respectively (see Figures 1A,B). A knot setter was usually required to tie the knots.

The aortic root was then exposed with stay sutures. The coronary buttons were constructed and retracted using stay sutures, and the remaining portion of aortic sinuses was removed. The anastomotic site of coronary buttons on the graft was determined with the heart filled and the graft distended. The left main coronary anastomosis (see Figure 1C) was then completed with 5–0 polypropylene followed by the right main (see Figure 1D). Sutures were started on the posterior aspect of the anastomosis. These suture lines were continuous, but they were intermittently tightened using a nerve hook to avoid the suture from becoming loose. A small autologous pericardial strip was placed between native coronary button and the graft.

The length of the graft was determined with the heart filled and the graft distended. The distal anastomosis of graft-native ascending aorta was completed in a running fashion using 4–0 polypropylene. These suture lines were continuous (see Figure 1E), but they were intermittently tied to avoid the suture from becoming loose. Hemostatic glue was placed around the suture line for additional hemostasis. A temporary epicardial ventricular pacing wire was placed on the inferior aspect of the right ventricle before releasing the cross-clamp. Carbon dioxide was infused into the operative field through the utility port at a flow of 0.5 L/min during the entire procedure. The left ventricle and the graft were de-aired with a venting needle in the root of the aorta (see Figure 1F) and under TEE guidance, and the cross-clamp was then removed.

Transesophageal echocardiography was used to meticulously evaluate ventricular function, prosthetic valve function, and the blood flow at the anastomoses of coronary arteries-graft. After discontinuing CPB and administering protamine, decannulation was performed. The purse-string sutures were tied and the femoral/axillary artery purse-string was reinforced with a 5–0 Prolene suture. After hemostasis was obtained, a pleural chest tube and a flexible pericardial tube were placed. The chest tubes along with the pacing wire were exited through the utility port. An On-Q pain relief system (I-Flow Corporation, Lake Forest, CA, United States) was inserted in each patient to provide pain relief. Two catheters were placed in the interspace to deliver 0.25% bupivicaine for 72 h. The incision was closed in the usual fashion.

### Statistical Analysis

Statistical analysis was performed with the SPSS statistical package version 22.0 (SPSS Inc., Chicago, IL, United States). Categorical data were expressed as frequency distributions and single percentages and were compared between groups using *Fisher’s* exact test if the expected frequency was <5 or the *chi-square* test. Normally distributed continuous variables were expressed as the mean ± standard deviation and were compared between groups using an independent-samples *t*-test; non-normally distributed continuous variables were expressed as median and interquartile range (IQR) and were compared between groups with the *Wilcoxon* rank sum test. A two-sided *p*-value less than 0.05 was considered statistically significant.

## Results

### Study Population

During this study period, a total of 481 Bentall procedures were carried out at our institution, including 450 full sternotomy procedures, 16 ministernotomy, and 15 right minithoracotomy. Fifteen consecutive patients who underwent right minithoracotomy Bentall procedure were identified. There were 15 male patients with the age ranging 19.0–69.0 years with a median of 58.0 years. This series included 2 patients with a history of percutaneous coronary intervention without requiring bypass grafting determined by preoperative invasive coronary angiography. Six patients were identified as Marfan’s syndrome, and another 8 had congenital bicuspid aortic valve. Severe aortic insufficiency was reported in all 15 patients, of whom 3 were diagnosed with concomitant severe aortic stenosis. The diameter of the aortic sinus ranged from 47.0 to 80.0 mm with a median of 49.5 mm, with no significant dilatation of the ascending aorta (median, 40.5 mm). The median left ventricular ejection fraction was 61.5%, with the median left ventricular endo-diastolic diameter of 60.5 mm (ranging 54.0–64.0 mm). Notably, one patient with Marfan’s syndrome had a previous mitral valve repair *via* a full median sternotomy approach. The detailed baseline characteristics are shown in Table 1.

**TABLE 1:** Baseline characteristics.

Variable	Value
**Number of patients**	15
Age (median, IQR; years)	58.0(51.0−64.3)
Gender (Males)	15(100%)
Body mass index (median, IQR; kg/m^2^)	23.6(21.8−26.2)
Recent smoking	3(20.0%)
**Concomitant diseases**	
Hypertension	7(46.7%)
Coronary artery disease	2(13.3%)
Spinal scoliosis	1(6.7%)
**Preoperative cardiac status**	
Previous cardiac surgery	1(6.7%)
**NYHA functional class**	
II	5(33.3%)
III	9(60.0%)
IV	1(6.7%)
LVEDD (median, IQR; mm)	60.5(54.0−64.0)
LVESD (median, IQR; mm)	39.5(34.3−43.3)
LVEF (median, IQR;%)	61.5(58.0−65.3)
**Aortic valve and root diseases**	
Etiology	
Marfan’s syndrome	6(40.0%)
Bicuspid aortic valve malformation	8(53.3%)
**Aortic valve pathology**	
Severe insufficiency	12(80.0%)
Severe insufficiency with severe stenosis	3(20.0%)
Diameter of aortic sinus (median, IQR; mm)	49.5(47.8−59.0)
Diameter of ascending aorta (median, IQR; mm)	40.5(38.0−42.0)

*IQR, interquartile range; NYHA, New York Heart Association (classification); LVEDD, left ventricular endo-diastolic diameter; LVESD, left ventricular endo-systolic diameter; LVEF, left ventricular ejection fraction.*

### Surgical Variables and Intraoperative Outcomes

All 15 patients underwent mini-invasive Bentall procedure *via* the right anterior thoracotomy approach (see Figure 1). The aortic cross-clamp time ranged from 80.0 to 105.0 min with a median of 95.0 min. The median duration of cardiopulmonary bypass was 138.5 (IQR, 130.5–163.5) min. Types and sizes of prosthetic valves are listed in Table 2. Adequate surgical exposure was obtained in all 15 patients, and there were no conversions to sternotomy performed. Intraoperative TEE immediately after the discontinuation of CPB showed that no instances of periprosthetic fistula were recorded.

**TABLE 2:** Procedure characteristics.

Variable	Value
CPB time (median, IQR; min)	138.5 (130.5–163.5)
ACC time (median, IQR; min)	95.0 (85.5–98.8)
Mechanical aortic valved graft	8 (53.3%)
Size of mechanical prosthetic valve	
23	1
25	4
27	3
Bioprosthetic valve plus graft	7 (46.7%)
**Size of bioprosthetic valve**	
23	2
25	5
**Size of graft**	
28	2
30	5

*CPB, cardiopulmonary bypass; IQR, interquartile range; ACC, aortic cross-clamping.*

One patient with Marfan’s syndrome without a previous cardiac procedure underwent an immediate repeat operation due to the left coronary kinking determined by TEE examination before the discontinuation of CPB. This was the first right minithoracotomy Bentall procedure in this center. After re-block perfusion with myocardial protection using the modified *Del Nido* cardioplegia solution, the anastomosis of native left coronary button-graft was removed, further dissociation the left main coronary from the surrounding tissue was performed, and the anastomosis of native left main coronary button-graft was then reconstructed. Afterward, successful weaning off bypass was recorded.

### In-Hospital Outcomes

The mechanical ventilation time ranged from 7.0 to 46.0 h with a median of 12.5 h. The median drainage volume in the first 24 h following surgery was 200.0 (IQR, 117.0–552.5) mL, with no redo for bleeding. Three (20.0%) patients received blood transfusion. There were no significant postoperative complications noted, including surgical death, low cardiac output, acute kidney injury requiring hemodialysis, and cerebrovascular accidents (Table 3). The length of ICU stay ranged from 0.5 to 4.0 days with a median of 1.5 days. All 15 patients were discharged smoothly, with the mean length of postoperative hospital stay of 5.8 ± 1.2 days.

**TABLE 3:** Perioperative and follow-up results.

Variables	Value
**Intraoperative**	
Number of patients	15
Immediate repeat operation	1 (6.7%)
**In-hospital**	
Number of patients	15
Surgical death	0
Blood transfusion	3 (20.0%)
^#^ Drainage volume (median, IQR; ml)	200.0 (117.0–552.5)
Mechanical ventilation time (median, IQR; hours)	12.5 (11.0–25.0)
Length of ICU stay (median, IQR; days)	1.5 (1.0–3.0)
Postoperative hospital stay (days)	5.8 ± 1.2
**Follow-up**	
Number of patients	15 (100%)
Duration of follow-up (median, IQR; months)	8.0 (6.0–10.0)
Survival	15 (100%)
Reoperation	0
**NYHA class at the latest follow-up**	
I	11 (73.3%)
II	4 (26.7%)

*IQR, interquartile range; ICU, intensive care unit.*

*^#^ Drainage volume, the total amount of drainage in the first 24 h following surgery.*

### Follow-Up Results

**FIGURE 2:**
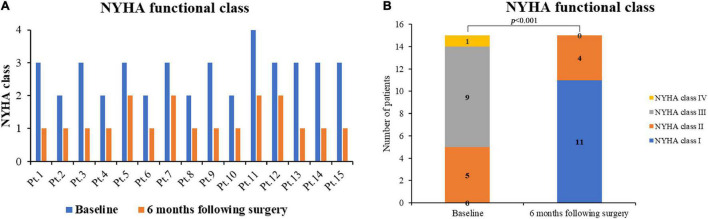
NYHA functional class prior to surgery and 6 months following surgery. **(A)** NYHA class preoperatively and postoperatively per patient; **(B)** NYHA class (baseline vs. 6-m after surgery, *p* < 0.001). NHYA, New York Heart Association; Pt., patient.

**FIGURE 3:**
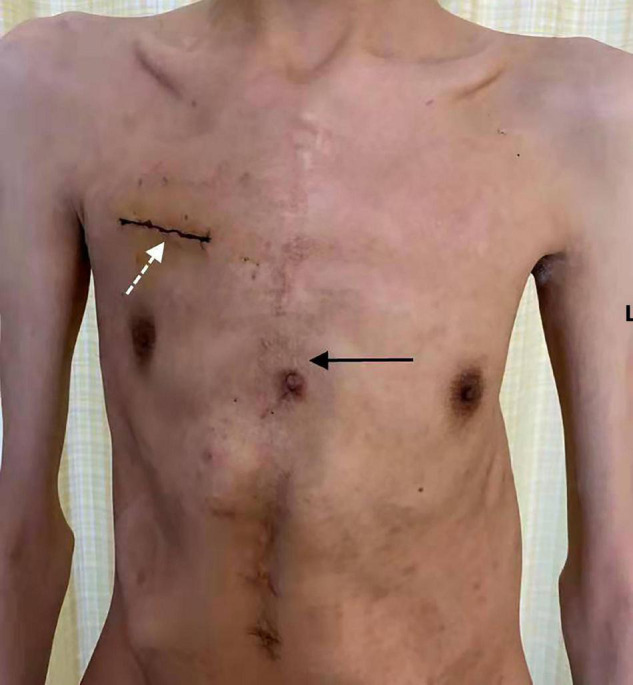
Skin incision. The image shows a skin incision in the right minithoracotomy Bentall procedure (where the white dotted arrow is pointing) and a skin incision in previous mitral valve repair *via* full median sternotomy (where the black arrow with a solid line is pointing).

All 15 patients received a follow-up visit with a median duration of 8.0 (IQR, 6.0–10.0) months. The clinical symptoms resolved, and no death or re-intervention was recorded. The shortest follow-up period was 6 months. By 6 months following surgery, NYHA functional class significantly decreased from the preoperative value (*p* < 0.001), with no patients in class III or IV (Figure 2). The incision healed well (Figure 3), and no complications such as poor healing and infection were found. No cases of periprosthetic fistula or significant aortic valve or mitral regurgitation determined by TTE were recorded at follow-up.

## Discussion

With mini-invasive aortic valve surgery becoming more common, surgeons worldwide have added more complex pathology to their mini-invasive techniques to allow patients the benefit of not having to undergo a full sternotomy (9, 10). Various mini-invasive approaches, including right parasternal incision, upper T incision, upper J incision, S-shaped partial sternotomy, L incision, as well as Z-, I-, and C-shaped ministernotomy incisions, have been used for surgery of the aortic root and the ascending aorta (9). Much of the literature reviewed showed the feasibility and favorable results of mini-invasive techniques for complex aortic pathology involving some type of partial sternotomy. Previously, Byrne et al. (4) reported a series of 290 consecutive patients who underwent aortic valve, aortic root, and/or ascending aorta replacement *via* either a partial upper sternotomy (87%) or a parasternal (13%) approach. They found that this approach was feasible with an acceptable incidence of complications without compromising the surgical procedure. Afterward, Tabata et al. (11) performed a 5 years follow-up of 128 patients who had ascending aortic, arch, and root surgery *via* an upper ministernotomy, and emphasized that this approach for complex aortic pathology was feasible and achieved an excellent late outcome. Recently, Perrotta et al. (9) reviewed the literature on various mini-invasive approaches for aortic root and ascending aorta surgery, and found that not only was a mini-invasive technique for complex aortic pathology feasible, but there were significant advantages when compared to full sternotomy. A right anterior minithoracotomy approach, with no performing any type of sternotomy, may lead to less surgical trauma and better outcomes. Johnson et al. (8) in a retrospective review of seven patients, described elective Bentall procedure performed through a right minithoracotomy approach and received favorable outcomes. It’s worth noting that in that series, video guidance was used in all patients, automated suturing technology was applied in some cases, and total circulatory arrest was used in all cases of distal anastomosis. Recently, a case report (12) presented a right minithoracotomy technique for Bentall procedure with conventional instruments and suturing techniques. We have completed 15 right minithoracotomy Bentall procedures during the last 1 year and a half. To the best of our knowledge, this is probably the largest experience of adult patients undergoing the right minithoracotomy Bentall procedure published to date. In our series, conventional instruments and suturing techniques were used without total circulatory arrest for distal anastomosis. Neither rib disarticulation was done, nor was the right internal mammary artery sacrificed. In this series, shortened length of intensive care unit as well as hospital stay coincided with neither in-hospital death nor neurological complication nor bleeding complication, which suggested that the right minithoracotomy Bentall procedure may be performed safely with low morbidity and mortality. During a follow-up period of at least 6 months, all 15 patients survived without reoperation, and their NYHA functional class improved, with no periprosthetic fistula or significant aortic valve regurgitation found. Results indicated the safety and effect of the right minithoracotomy Bentall procedure.

The major disadvantage of the right minithoracotomy approach for Bentall procedure is the longer circulatory arrest time required compared with sternotomy approaches. The median CPB time in this series was 138.5 min, which is much shorter than the time of 202.9 ± 47.8 min in the case series by Johnson et al. (8). Similarly, the median cross-clamp time in this series was 95.0 min, which is meaningfully shorter than the mean cross-clamp time of 161.9 ± 32.1 min reported in their study (8). The reduction in the aortic cross-clamp time and CPB time may be due to the increasing experience with this technique and standardization of the operative steps. In this center, the right minithoracotomy AVR has become a common practice over the years with an annual volume of over 300 cases, and Bentall procedure through sternotomy was carried out about 300 cases per year. In this series including 15 right minithoracotomy Bentall procedures, 14 were carried out by Professor CSW and one by Professor HL. Professor CSW has completed Bentall procedure through sternotomy over 50 cases per year and right minithoracotomy AVR about 200 cases per year, and Professor HL has completed Bentall procedure through sternotomy over 20 cases per year and right minithoracotomy AVR of over 30 cases per year. We believed that the right minithoracotomy Bentall procedure should be performed by cardiac surgeons who have gained sufficient experience with right minithoracotomy AVR and who also had rich experience in Bentall procedure through sternotomy.

In our opinion, the right minithoracotomy Bentall procedure should be performed in carefully selected patients. In this initial clinical experience, selection criteria included patients with normal or near normal left ventricular ejection fraction, no significant coronary artery disease requiring bypass grafting or with concomitant other cardiac procedures, and aortic arch pathology requiring no greater than a hemiarch resection. We avoided patients with aortic dissection or intra-mural hematoma, or surgical procedures requiring aortic valve preservation. It was also preferable not to use this technique to operate in patients with peripheral vascular disease. A preoperative CT scan of the chest was performed in all patients to better delineate the anatomy as well as determine the presence and extent of calcification of the aorta. Patients with aortas positioned left of the midline on preoperative imaging were not good candidates for this approach. Patients were excluded from this approach if they had heavily calcified ascending aorta or porcelain aorta (13), thoracic deformity, anomalous origin of coronary artery, and poor pulmonary function. This approach was not recommended when patients underwent previous right thoracic surgery. Previous studies (14) reported that a prior sternotomy was a relative contraindication to this procedure. In fact, one patient in this series who had a history of mitral valve repair *via* a full median sternotomy underwent the right minithoracotomy Bentall procedure and received favorable results. We believed that a prior mitral valve procedure with a full median sternotomy was not a contraindication to the right minithoracotomy Bentall procedure, but a prior aortic valve procedure with or without ascending aortic procedure with a full median sternotomy may be a relative contraindication to this approach. In addition, patients or the family agreed to receive this rarely performed, mini-invasive approach for complex aortic root surgery, and signed the informed consent.

Notably, only a minority of patients (3.1%) who underwent Bentall procedure received the right minithoracotomy approach. There may be several reasons behind it. In this initial clinical experience, only extremely “healthy” patients (with a normal ejection fraction and having relatively few comorbidities) were chosen to undergo this approach, and only two experienced cardiac surgeons have carried out this procedure. The successful experience of Professor HL in carrying out the right minithoracotomy Bentall procedure suggested this technique may be adopted and reproduced, and it inspired more surgeons to use this sternal sparing approach for Bentall procedure. In fact, Doctor QJ has completed one right minithoracotomy Bentall procedure. The patient was discharged on postoperative day 5. The patient received routine follow-up at 1-month following surgery, was asymptomatic, and was carrying out his daily activities. This again suggested that the right minithoracotomy Bentall procedure may be adopted and reproduced. Patient concerns about safety and effect of this initial approach for Bentall surgery may also be a contributing factor. We believed that with the accumulation of experience and the increase of successful cases, more cardiac surgeons will carry out this procedure, and more patients will accept this approach.

In this center, a femoral rather than an axillary platform was utilized preferably to establish the CPB as small inner diameter of the axillary artery of Chinese patients made it difficult to cannulate with above 16 Fr arterial cannula. In this series, no retrograde iatrogenic aortic dissection associated with femoral artery cannulation was observed. Notably, pericardial stay sutures should be employed liberally in order to mobilize the aorta prior to the cross-clamp. The bottom jaw of the aortic cross clamp should be placed above the right pulmonary artery to provide more operating space. It was very important to perform an excellent anastomosis at each site and in particular coronary button anastomosis. A small autologous pericardial strip placing between native coronary button and the graft was introduced to reduce the risk of this complication. More critically, the left and right main coronary should be dissociated fully, a serious lesson for us, to avoid coronary kinking after anastomosis. *Via* the right anterior thoracotomy approach, the aortic root was replaced in all 15 patients, and the distal extent of reconstruction was above the level of transverse sinus and reached the level of the right pulmonary artery. Meticulous attention to maintaining hemostasis was critical in this procedure, because bleeding will be more difficult to control, particularly at the left and the right main coronary artery anastomoses. Multimodal analgesic therapy, including intercostal nerve blocks, peri-incisional lidocaine patches, and acetaminophen, may contribute to reducing narcotic pain medicine requirements and promoting early mobilization.

The current study was subject to the limitations inherent in a single-center, retrospective study design with a short follow-up. A control group including patients undergoing Bentall procedure *via* full or partial sternotomy was not established, and the number of patients undergoing the right minithoracotomy Bentall procedure was relatively small (15 patients), implying potential weakness of the results. Finally, the patients studied in this series had a normal ejection fraction and had relatively few comorbidities suggesting that the study results might not be generalizable to sicker patients undergoing this approach. As surgeon experience increases, selected sicker patients may be candidates for this approach.

## Conclusion

The present study demonstrated that the right minithoracotomy Bentall procedure may be performed safely with low morbidity and mortality. It should be considered as an option in carefully selected patients requiring aortic root replacement.

## Data Availability Statement

The original contributions presented in the study are included in the article/supplementary material, further inquiries can be directed to the corresponding authors.

## Ethics Statement

The studies involving human participants were reviewed and approved by the Ethics Committee of Zhongshan Hospital Fudan University. The patients/participants provided their written informed consent to participate in this study.

## Author Contributions

QJ, YW, and FL contributed equally in the data collection, statistical analysis and manuscript drafting. YY, JL, XS, ZY, and SP participated in data collection, patient follow-up and manuscript revision. CW and HL were responsible for the study design, manuscript revision and consultation. All authors contributed to the article and approved the submitted version.

## Conflict of Interest

The authors declare that the research was conducted in the absence of any commercial or financial relationships that could be construed as a potential conflict of interest.

## Publisher’s Note

All claims expressed in this article are solely those of the authors and do not necessarily represent those of their affiliated organizations, or those of the publisher, the editors and the reviewers. Any product that may be evaluated in this article, or claim that may be made by its manufacturer, is not guaranteed or endorsed by the publisher.
